# Understanding structure-guided variant effect predictions using 3D convolutional neural networks

**DOI:** 10.3389/fmolb.2023.1204157

**Published:** 2023-07-05

**Authors:** Gayatri Ramakrishnan, Coos Baakman, Stephan Heijl, Bas Vroling, Ragna van Horck, Jeffrey Hiraki, Li C. Xue, Martijn A. Huynen

**Affiliations:** ^1^ Department of Medical Biosciences, Radboud University Medical Center, Nijmegen, Netherlands; ^2^ Bio-Prodict, Nijmegen, Netherlands; ^3^ Vartion, Malden, Netherlands

**Keywords:** protein structure, 3D CNN, missense variant, machine learning, gain-of-function, loss-of-function

## Abstract

Predicting pathogenicity of missense variants in molecular diagnostics remains a challenge despite the available wealth of data, such as evolutionary information, and the wealth of tools to integrate that data. We describe DeepRank-Mut, a configurable framework designed to extract and learn from physicochemically relevant features of amino acids surrounding missense variants in 3D space. For each variant, various atomic and residue-level features are extracted from its structural environment, including sequence conservation scores of the surrounding amino acids, and stored in multi-channel 3D voxel grids which are then used to train a 3D convolutional neural network (3D-CNN). The resultant model gives a probabilistic estimate of whether a given input variant is disease-causing or benign. We find that the performance of our 3D-CNN model, on independent test datasets, is comparable to other widely used resources which also combine sequence and structural features. Based on the 10-fold cross-validation experiments, we achieve an average accuracy of 0.77 on the independent test datasets. We discuss the contribution of the variant neighborhood in the model’s predictive power, in addition to the impact of individual features on the model’s performance. Two key features: evolutionary information of residues in the variant neighborhood and their solvent accessibilities were observed to influence the predictions. We also highlight how predictions are impacted by the underlying disease mechanisms of missense mutations and offer insights into understanding these to improve pathogenicity predictions. Our study presents aspects to take into consideration when adopting deep learning approaches for protein structure-guided pathogenicity predictions.

## 1 Introduction

Numerous Mendelian diseases can be attributed to alterations in the coding regions of the DNA, i.e., missense variants (Kryukov et al., 2007). With rapid advances in sequencing technologies, the ease and ability to map a person’s complete genome has dramatically aided in obtaining genetic diagnosis. Nevertheless, only a small fraction of the missense mutations is pathogenic (Lek et al., 2016) and for the majority of missense variants it is not clear whether the phenotypic outcome is pathogenic or neutral. Such variants are coined “variants of uncertain significance” (VUS). Evidently, identifying and comprehending the functional effects of missense variants is of critical importance, not only to understand the etiology of the disease but also towards development of treatment regimens.

Significant advances have been made in the development of variant effect predictors that largely rely on evolutionary conservation, which is a strong signal for predicting pathogenicity. Such evolutionary cues in combination with physicochemical properties of amino acids form the base framework of several state-of-the-art techniques including SIFT (Ng and Henikoff, 2003), PolyPhen2 (Adzhubei et al., 2010), CADD (Kircher et al., 2014), and MutPred (Li et al., 2009). Although evolutionary information holds value in predicting pathogenicity, it does not provide mechanistic understanding. The mechanisms of the pathogenicity of missense variants are often attributable to perturbations in conformational and functional properties of three-dimensional structures (Wang and Moult, 2001; Iqbal et al., 2020), which can contribute to our understanding of the underlying molecular pathology. Several studies have thus incorporated features that leverage structural properties (Venselaar et al., 2010; Capriotti and Altman, 2011; Ittisoponpisan et al., 2019; Laskowski et al., 2020), protein dynamics (Ponzoni et al., 2020), protein-protein interaction networks (Yates et al., 2014), and protein structural stability (Ancien et al., 2018), to improve pathogenicity predictions on top of what can be achieved with sequence conservations. In the absence of experimental structural information, context-dependent sequence-based models have the potential to accurately capture intra-protein 3D contacts, i.e., via evolutionarily coupled residues (Morcos et al., 2011; Marks et al., 2012; Hopf et al., 2014). Utility of such models has shown reasonable improvement in distinguishing pathogenic missense variants from benign ones (Feinauer and Weigt, 2017; Hopf et al., 2017). A complete list of available resources and tools for variant effect prediction and their benchmark evaluation studies has been published elsewhere (Liu et al., 2011; Livesey and Marsh, 2022). Despite the significant advances, the challenge of distinguishing pathogenic variants from benign ones remains elusive with most methods exhibiting a wide spectrum of performances on different test datasets (Niroula and Vihinen, 2019; Livesey and Marsh, 2020).

Most knowledge-driven approaches that employ machine learning (ML) classifiers rely on various handcrafted features to predict variant effects, which could be time-consuming and laborious. This is compounded by heterogeneity in feature attributes that can pose challenges in data integration (Bagley and Altman, 1995). Deep learning accelerated approaches can help overcome such limitations. CNNs have gained prominence in the last decade due to their ability to automatically capture patterns from input data as well as the hierarchical representations therein (Krizhevsky et al., 2012), enabling them to capture relationships between different features. This aspect is particularly useful for analyzing high dimensional data such as protein structures.

Recent efforts have demonstrated the use of 3D-CNNs in exploiting protein structure data for several applications including the prediction of amino acids compatible with protein microenvironments (Torng and Altman, 2017; Pun et al., 2022), identification of novel gain-of-function mutations (Shroff et al., 2020), and the prediction of mutation-induced changes in protein stability (Li et al., 2020). We introduce DeepRank-Mut, a configurable 3D-CNN framework that predicts pathogenicity of missense variants using wildtype structural microenvironment surrounding the variants in 3D space. The base framework is derived from its parent DeepRank that distinguishes and ranks biologically relevant protein-protein interactions from those that arise due to crystallographic artifacts (Renaud et al., 2021). The underlying premise of our approach is that the functional outcome of any missense variant is often reflected in the properties of amino acids in the variant neighborhood, in addition to the properties of the variant amino acid itself. Our approach is similar to the method devised by Torng and Altman (2017), which, given a site, predicts the amino acids compatible with that specified site based on the surrounding protein microenvironment. In contrast, we train our model explicitly to learn label-specific (benign or pathogenic) features/patterns in the variant neighborhood. Given a missense variant, we first obtain the associated 3D protein structure, either from the protein itself or from a homolog, and calculate features including surface geometry, empirical energies, and atomic densities, in addition to the sequence conservation scores for the mutated site as well as the residues in its neighborhood. These features are mapped onto 3D grids parameterized using properties of the constituent atoms, followed by data augmentation to enrich the input dataset. We then use the power of 3D-CNNs to automatically discern spatially proximal features within these representations.

DeepRank-Mut achieves a performance comparable to techniques that efficiently combine sequence and structure-based features. We analyze the contribution of each of the features to the model’s predictive ability, as well as how the neighborhood contributes to the performance. To better understand predictor accuracy, we explore underlying mechanisms of pathogenic mutations and show that the features identify autosomal recessive mutations better than autosomal dominant mutations. We discuss the overall generalizability of our method and provide avenues for better 3D-based missense variant prioritization.

## 2 Methods

### 2.1 Datasets

A total of 193,714 missense variants (164,574 benign, 29,140 disease-causing) were collected from ClinVar (Landrum et al., 2018), gnomAD (Karczewski et al., 2020) and Dutch genome diagnostic laboratories (VKGL, 2019), which could be linked to protein structures, either directly or through homology with a sequence identity cut-off of 40%. This cutoff was selected based on previous research that suggests that a 40% identity corresponds to a good likelihood of functional equivalence (Pearson, 2013). Missense variants were mapped onto protein structures using 3DM systems as a guide (Kuipers et al., 2010). Independent test datasets were obtained from studies based on BRCA1 (Findlay et al., 2018), Gunning et al. (2021) and the InSIGHT database (Thompson et al., 2014). This resulted in a total of 217,679 missense variants that could reliably be mapped onto 57,551 structures; 25,856 structures were mapped to 40,369 pathogenic variants, and 31,695 structures were mapped to 177,310 benign variants. It should be noted that, at this stage, the structures are mapped regardless of the experimental method used for their determination. Missense variants from ClinVar were incorporated if they had a review status of at least one star, excluding those with conflicting interpretations. “Benign” and “Likely benign” ClinVar variants were included and categorized as benign, while “Pathogenic” and “Likely pathogenic” variants were incorporated and classified as pathogenic. The gnomAD variants with a minor allele frequency higher than 0.1% were selected and labeled as benign.

Our in-house database, HSSP (Touw et al., 2015) was consulted to obtain structure-based sequence alignments. Position-specific scoring matrices (PSSMs) were constructed for the alignments using PSI-BLAST (Altschul et al., 1997) with single iteration. Each of the PSSMs were then mapped back onto their respective structures using the PSSMGen package (https://github.com/DeepRank/PSSMGen).

### 2.2 Data pre-processing

#### 2.2.1 Feature calculation and voxelization of the neighborhood

**FIGURE 1:**
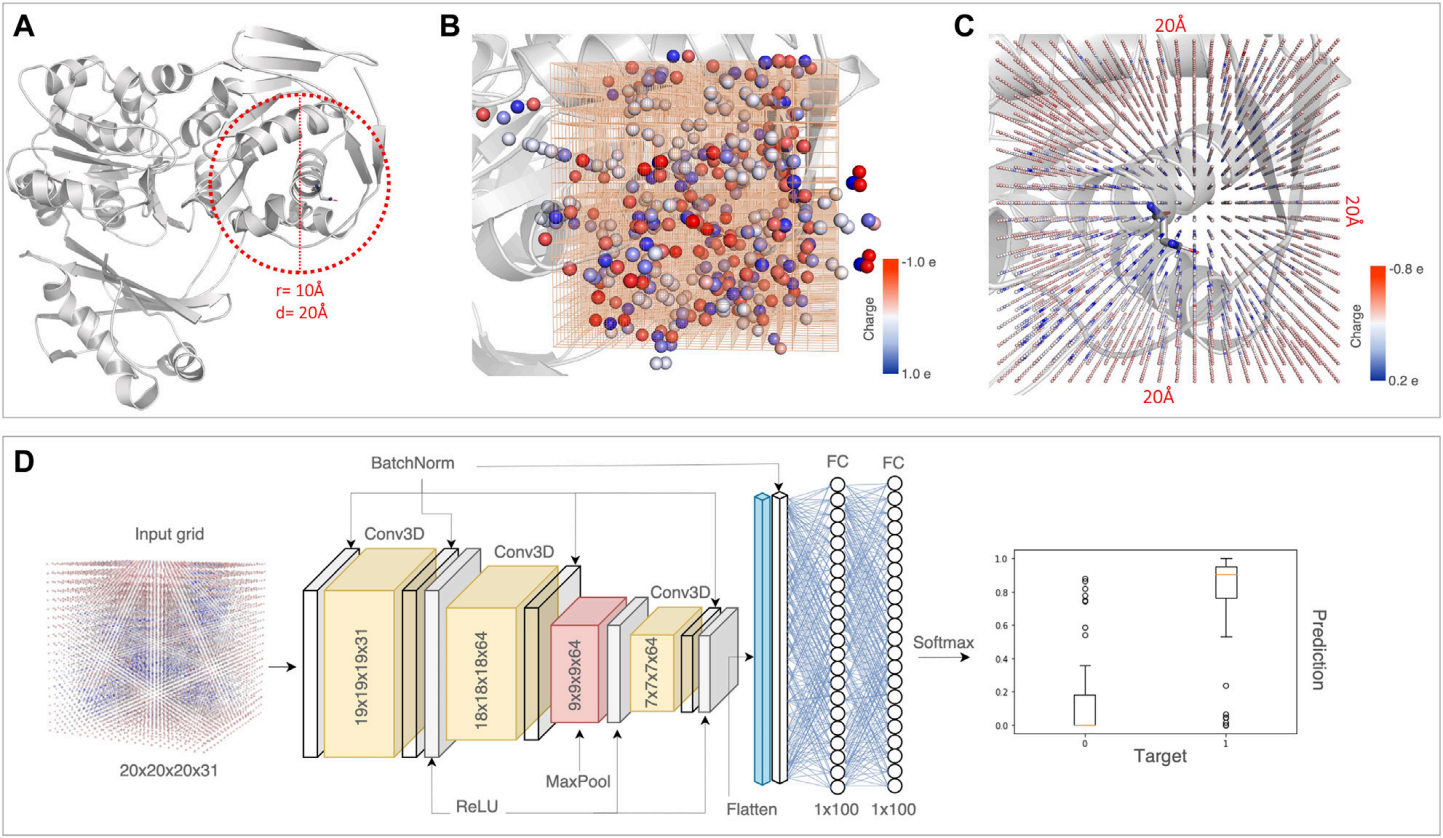
A schematic of the DeepRank-Mut framework. **(A)** The first step includes extraction of the variant environment, where residues within a radius of 10Å (diameter of 20Å) around the variant are drawn. As an example case, the crystal structure of phosphoglucomutase (PDB: 1C4G) with the missense variant Asn37 is depicted. This is followed by the feature calculation step where structural properties and PSSM scores are computed for the variant site and the residues in its environment. All features are localized on atoms as illustrated in **(B)**. For simplicity, one structural property (charge), localized on atoms, is shown. A 3D grid of size 20 × 20 × 20 is centered at the C*α* atom of the residue at variant site, discretized into voxels of 1Å. **(C)** Each of the features calculated are normalized using standardization and then mapped onto the grid using a Gaussian function. For simplicity, the Gaussian mapping of one feature, i.e., charge for all atoms within a 20Å box is depicted. In principle, a total of 31 calculated features are mapped to the 3D grid for a given variant. **(D)** This 3D grid with mapped features of shape (20, 20, 20, 31) serves as an input for the 3D-CNN network. The final classification score takes a value between 0 and 1 for each class (benign and pathogenic).

We use protein crystal structures of resolution better than 3Å in our study, as these provide details at the atomic level with high certainty (Zardecki et al., 2022). Consequently, variants that are mapped to structures solved using methods other than X-ray crystallography, such as NMR or cryo-EM, are excluded. For ease in data handling, we mapped each missense variant to a maximum of three crystal structures of the most similar sequences. For each variant mapped to a crystal structure, we first extract the local neighborhood with a radius of 10Å around the variant, which typically serves as a distance beyond which the strength of long-range non-bonded interaction energies gradually weakens (Pincus and Scheraga, 1977). We include residues whose atoms fall within this radius to obtain residue-based features. This is followed by calculation of atomic features such as densities and charges for the wildtype amino acid and the residues in its microenvironment. Pairwise Coulomb and van der Waals potentials are calculated between atoms of the wildtype residue and the residues in the neighborhood. For a given atom, these features are defined as the sum of all pairwise potentials between the atom and its contact atoms. Bonded pairs, i.e., pairs of atoms separated by up to 2 bonds are excluded from this measure. The atomic densities, charges and non-bonded energies are based on the OPLS force field (Jorgensen and Tirado-Rives, 1988), calculated in the same manner as in the parent DeepRank (Renaud et al., 2021) (see Supplementary Methods). Solvent accessible surface area (SASA) is calculated using FreeSASA (v2.0.3) (Mitternacht, 2016). Water molecules in protein structures, when present, are not included in the analysis. In addition to the PSSM obtained for the wildtype and variant amino acids, we also include the PSSM profile for the residues in the variant microenvironment. Such residue-based feature values are assigned to the residue’s constituent atoms. All feature values are localized on atoms, to be subsequently mapped on a 3D grid (see Figure 1); only those atoms that lie within 10Å radius of the variant are considered. At this stage, it should be noted that some structures in the PDB database may contain missing residues that fall within the variant environment radius, leading to errors in the feature mapping step. Such molecules are thus, excluded from the dataset.

We construct a 3D grid of size 20Å × 20Å × 20Å centered at the C*α* atom of the amino acid at the variant site. This 20Å box is divided into voxels of 1Å, parameterized with 31 physicochemical property channels (Table 1). The properties are mapped on a 3D grid using Gaussian functions to approximate atom connectivity, as demonstrated previously in the parent DeepRank framework (Renaud et al., 2021). The contribution (*w*_k_) of an atom *k* to a given grid point is determined based on Gaussian distance dependence, i.e., the contribution decreases with increasing distance between the atom and the grid point. This is given by the equation:

$$w_{k}\left(r\right)=v_{k}\exp\left({\left\|r-r_{k}\right\|}^{2}\;/2\sigma^{2}\right)$$ (1)

where *v*_*k*_ is the feature value, *r* denotes position of the grid point and *r*_*k*_ denotes atomic coordinates (x, y, z). The standard deviation *σ* denotes the van der Waals radius of the associated atom.

**TABLE 1:** List of features calculated for the residue at the mutation site and the residues in its neighborhood.

Features	Number of channels
Atomic densities (C, N, O, S)	4
Atomic charges	1
Solvent accessibility	1
Coulomb potential	1
van der Waals potential	1
Wildtype score: PSSM	1
Variant score: PSSM	1
Information content (PSSM)	1
PSSM profile	20
Total	31

All features, including residue-level features such as sequence conservation scores, are localized on atoms. The two sequence-based features (wildtype and variant probability) are mapped to the atoms of a given wildtype residue.

The feature maps are stacked to create a tensor of shape (20, 20, 20, 31) that then serves as an input to the neural network. We also normalize features of the input data using standardization prior to training. To optimize for speed and efficient handling of large volumes of data, we developed a distributed data preprocessing framework with GPU support, which enabled faster preprocessing times and scalability during numerous iterations of experiments (see Supplementary Methods, Supplementary Figure S1).

#### 2.2.2 Data augmentation

Prior to the training step, we enrich each of the input 3D grids using data augmentation where a given grid is randomly rotated around its center, and features are mapped onto the grid subsequently. Such a strategy has been shown to improve the performance of CNNs (Shorten and Khoshgoftaar, 2019). For the current study, we used 5 augmentations based on hyper parameter tuning experiments (Supplementary Figure S3). We did not experiment with a higher number of augmentations due to the infeasible computational costs involved.

### 2.3 Network architecture

The network used in our study includes a sequential organization of three 3D convolutional layers, alternating with one 3D max pooling layer followed by two fully connected layers (Figure 1). We include batch normalization layers, in addition to dropout layers between the fully connected layers to regularize the model. Details of the architecture are provided in Table 2 and the complete schema is provided in Supplementary Figure S2. Each 3D convolution layer comprises a set of learnable filters that traverse the input space (depth, height and width) with a stride of 1, capturing local spatial patterns in the variant environment. The output from convolution operations, i.e., the computed feature maps are transformed by a rectified linear activation function (ReLU), which allows the network to identify and extract meaningful spatial features. This is followed by dimension reduction using max pooling operation and a final 3D convolutional layer with ReLU. The transformed output is then flattened to a one-dimensional vector that serves as an input to two fully connected layers. The two final layers integrate the features and apply a set of weights that are optimized during the training step to map extracted features to target classes. The output is then passed through the softmax function which provides the final classification score, a probability estimate between 0 and 1, each for benign and pathogenic classes.

**TABLE 2:** Network architecture used in DeepRank-Mut.

Layer	Size	Output shape
Batch normalization layer 1	Input	20 × 20 × 20 × 31
3D convolutional layer 1	20 × 20 × 20, 31 filters, kernel size = 2, stride = 1	19 × 19 × 19 × 31
Batch normalization layer 2		19 × 19 × 19 × 31
3D convolutional layer 2	19 × 19 × 19, 64 filters, kernel size = 2, stride = 1	18 × 18 × 18 × 64
Batch normalization layer 3		18 × 18 × 18 × 64
3D max pooling layer	Stride = 2	9 × 9 × 9 × 64
3D convolutional layer 3	9 × 9 × 9, 64 filters, kernel size = 3, stride = 1	7 × 7 × 7 × 64
Batch normalization layer 4		7 × 7 × 7 × 64
Flatten	7 × 7 × 7 × 64	21,952
Batch normalization layer 5		21,952
Fully connected layer 1	21,952 × 100 neurons	100 neurons
Dropout (*p* = 0.5)		
Fully connected layer 2	100 × 100 neurons	100 neurons
Dropout (*p* = 0.5)		
Softmax	100 × 2	2 scores (benign, pathogenic)

### 2.4 Training

We performed 10-fold cross validation experiments while ensuring that the missense variants in the training and validation sets are from different proteins, to avoid type 1 circularity in predictions (Heijl et al., 2020). The test dataset included missense variants independent from the 10-fold training and validation sets. Most genetic variation is neutral, and it is therefore rather common to observe a higher number of benign variants than pathogenic variants in the training data, which has the potential to bias training and performance. We thus constructed balanced subsets of randomly sampled benign and pathogenic missense variants for each of the 10-fold runs. For efficient memory handling, we employed training in mini-batches of 256 variant instances which amounted to ∼1,200 mini-batches per epoch. An epoch refers to a single pass through the complete training data during which the model weights are adjusted to minimize the error between predicted and true label for each input. With the input dataset, one epoch in our approach referred to one pass through more than 280,000 variant instances. We used the AdamW optimizer (Loshchilov and Hutter, 2019) with a learning rate of 0.001 and weight decay of 0.005 to train our model for 10 epochs. We used cross entropy loss during training, which attempts to minimize the differences in probability distributions between predicted and ground truth labels by adjusting weights. A dropout rate of 0.5 was used to regularize the model. The hyperparameters including number of convolutional layers, number of max pooling layers, grid size, were optimized based on performance on validation set across 10 folds, starting from default parameters of the parent DeepRank.

### 2.5 Evaluation metrics

Two metrics, Matthews Correlation Coefficient (MCC) and accuracy, were used to evaluate the performance of DeepRank-Mut. The primary metric used was MCC, as it offers a reliable statistical measure by taking all four categories-true positives (TP), true negatives (TN), false positives (FP), and false negatives (FN) into account, proportional to the size of the binary classes (Eq. 2). The usefulness of MCC over accuracy or F1 scores for binary classification has been demonstrated previously (Chicco and Jurman, 2020).

$$MCC=\frac{TP\times TN-FP\times FN}{\sqrt{\left(TP+FP\right)\times \left(TP+FN\right)\times \left(TN+FP\right)\times \left(TN+FN\right)}}$$ (2)

For comparative evaluation with popular state-of-the-art variant effect predictors, we used precomputed pathogenicity prediction scores of 8 algorithms from dbNSFP v4.3 database (Liu et al., 2020; 2011), including SIFT4G (Vaser et al., 2016), PolyPhen2 (Adzhubei et al., 2010), MutationTaster (Schwarz et al., 2014), MutationAssessor (Reva et al., 2011), FATHMM (Shihab et al., 2013), VEST4 (Carter et al., 2013), PROVEAN (Choi and Chan, 2015) and MutPred (Li et al., 2009), as well as prediction scores from Helix (Vroling and Heijl, 2021). Where available, we used “converted rankscores” from dbNSFP to ensure that a higher score always indicated higher likelihood of pathogenicity. We excluded meta predictors from this comparison, as well as those that combine annotations from other tools, to account for methods that rely on first principles to predict functional effects of missense variants.

## 3 Results

### 3.1 Overview of the datasets and DeepRank-Mut

Training, validation, and test sets are often generated using a simple random split. However, this can result in over fitting and misleading results due to data leakage between the training and evaluation sets (Heijl et al., 2020). Data splitting at the level of proteins or genes, where training sets never include any data samples from proteins that occur in the validation or test set is used to mitigate this. We split our dataset into 10 pairs of training and test sets, each containing 90% and 10% of the full dataset, respectively, allowing 10-fold cross validation on the full dataset. Independent test sets were gathered from three studies, as described in methods, to aid in the final assessment of the tool’s performance. These test sets have been selected as they not only cover genes in-depth (Thompson et al., 2014; Findlay et al., 2018) but are also aimed at benchmarking pathogenicity predictors specifically (Gunning et al., 2021).

After splitting the data, the balanced subsets of randomly sampled benign and pathogenic variants, each mapped to at most three structures, comprised a total of ∼50,000 instances in the training set, ∼4,700 instances in validation and 6,571 in the test set, per fold. The test set was kept identical across all cross-validation folds for an unbiased evaluation of the model.

DeepRank-Mut retains its modularity in implementing data pre-processing steps and training the deep neural network, similar to its parent DeepRank (Renaud et al., 2021). It allows for flexibility in tasks including feature calculations, setting the grid size and grid resolution, data augmentation, as well as optimizing hyperparameters of the neural network. The base requirements of DeepRank-Mut include a dataset of variants with labels (benign or pathogenic), a dataset of variant-structure maps where each variant is linked to a 3D structure (either experimentally determined or evolutionarily related), a dataset of 3D structures and an optional dataset of PSSM profiles derived for each structure. As detailed in the methods, the framework computes physicochemical properties of the amino acid at the variant site as well as its environment within a radius of 10Å, followed by voxelization to encode the atomic neighborhood of residues (Figures 1A, B). Our approach relies on leveraging local properties of sites characteristic of benign or pathogenic variants, as pathogenic variants generally tend to occur in regions important for structural/functional integrity of the protein (Iqbal et al., 2020), like its hydrophobic core. We thus compute a total of 31 features (Table 1), encompassing structural and sequence-based properties, for the residue at the variant site and residues spatially proximal to it. The computed features are mapped to a 3D grid where each voxel is parameterized with the feature channels (Figure 1C), which is then followed by data augmentation. As a given variant environment can differ in orientation within or across proteins, the data augmentation step accounts for rotational invariance, thereby improving the model’s robustness to variations in input data (Supplementary Figure S3). From our dataset of structures and missense variants, we generated ∼300,000 augmented grids per fold dataset, which were used as input to 3D-CNN (Figure 1D). Each augmented 3D grid is treated as a separate variant instance, thus our model outputs 6 predictions per missense variant (origin grid +5 augmented grids) which are averaged to give one final classification score.

### 3.2 Overall performance

**FIGURE 2:**
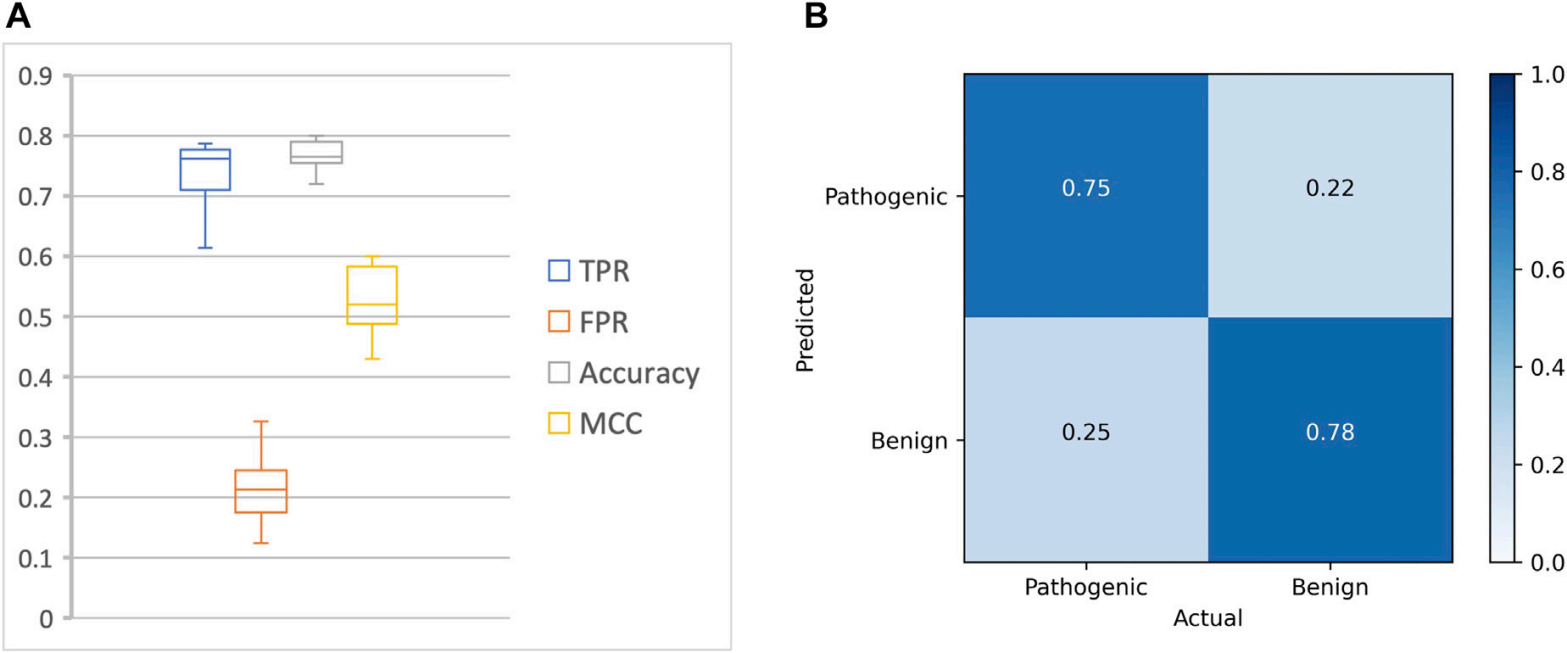
Overall performance of DeepRank-Mut. **(A)** The performance metrics of DeepRank-Mut on test sets across 10 folds are depicted as boxplots for true positive rate (TPR), false positive rate (FPR), accuracy and MCC. **(B)** Confusion matrix depicting the average of TP, FP, FN, and TN across 10 folds.

Our approach achieved a mean accuracy of 0.77 and an average MCC score of 0.52 across the test datasets, with an average sensitivity (true positive rate) of 0.75 and an average specificity (true negative rate) of 0.78 (Figure 2; Table 3).

**TABLE 3:** Details of the performance metrics of DeepRank-Mut on test sets across 10 folds.

Fold	TPR	FPR	Accuracy	MCC
1	0.613	0.142	0.78	0.49
2	0.765	0.241	0.76	0.52
3	0.70	0.124	0.79	0.59
4	0.768	0.258	0.76	0.51
5	0.777	0.24	0.77	0.52
6	0.787	0.188	0.80	0.60
7	0.713	0.235	0.74	0.48
8	0.777	0.191	0.79	0.58
9	0.758	0.326	0.72	0.43
10	0.716	0.186	0.76	0.53
Average	0.737	0.213	0.77	0.52

#### 3.2.1 Impact of individual features and the variant environment on the performance

**FIGURE 3:**
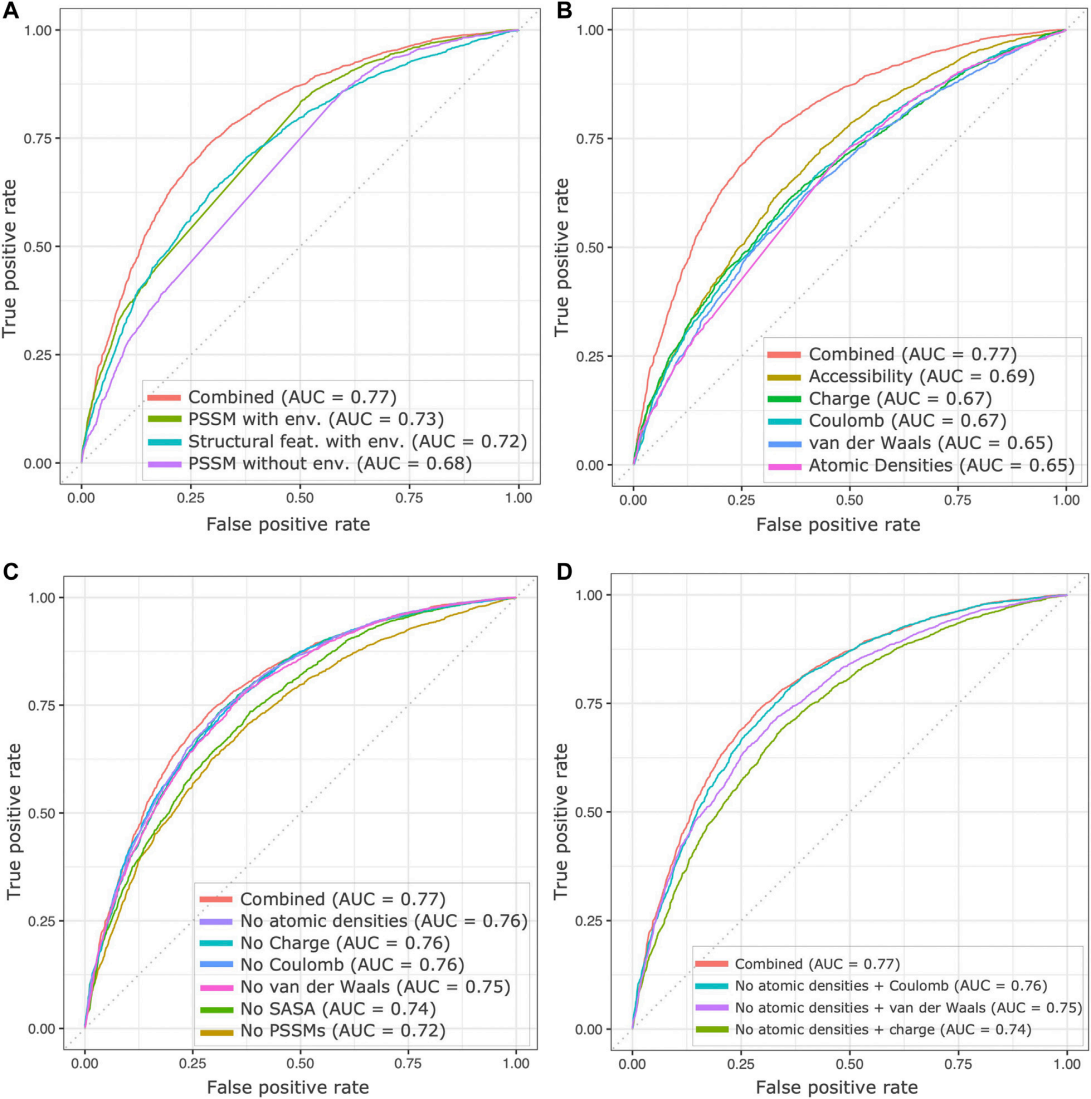
Contribution of the variant neighborhood and features to the model’s predictive ability. **(A)** Receiver operating characteristic (ROC) curves are drawn from scores generated by the complete model (red) and by models separately trained only on the PSSM profile of residues in the neighborhood (green), only on the structural features from the neighborhood (cyan) and PSSM of mutation site alone (purple). The AUC values obtained illustrate the value of using 3D neighborhood information in the predictions. **(B)** ROC curves are drawn using scores generated by models separately trained on the individual structural features. **(C)** ROC curves for leave-one-feature-out analysis are drawn using scores from models trained without a specific feature. Model trained without PSSMs (beige) and the model trained on structural features from neighborhood (cyan) in the first panel **(A)** are identical. **(D)** ROC curves are drawn for scores from models where redundant features are removed. For consistency the model trained on all 31 features is included in all panels. Total *n* = 6,571 instances, 3,804 pathogenic and 2,767 benign.

To investigate the contribution of neighborhood in the predictor accuracies, we compared the performance of our 3D-CNN model trained on all features to those trained separately on-a) PSSM features, b) structural features, c) variant site-specific PSSMs (Figure 3A). The model trained on PSSM features included PSSMs for the residues in the 3D neighborhood as well as the scores for wildtype and variant amino acids, while the model trained on variant site-specific PSSMs was devoid of the neighborhood profile. As illustrated in the figure, the features derived from the neighborhood, in the 3D context, seemingly hold more information than the site-specific features. This aspect was also observed during hyperparameter tuning experiments, where a range of different sizes of 3D grids were tested to find the optimal grid size. Models with smaller variant neighborhoods (grid sizes = 7Å, 8Å) performed poorly on validation sets as compared to the models with grid size of 15Å and 20Å (Supplementary Figure S4). It has been reported earlier that the atomic details do not provide significant information for local protein environments beyond a 20Å cutoff (Bagley and Altman, 1995). An optimal grid size of 20Å was thus chosen for all experiments. Additionally, we investigated the apparent contribution of individual structural features in prediction accuracies, as illustrated in Figure 3B. We note that solvent accessibility of residues has the most predictive capacity amongst all structural features. Residues buried in the hydrophobic core of the protein are often associated with pathogenicity, while solvent-exposed missense variants are often found to be enriched in populations, as also exemplified by Iqbal et al. (2020).

Additionally, we also performed leave-one-feature-out analysis to assess redundancy in our feature selection. Figure 3C illustrates similarity in ROC curves of models trained without pairwise potentials (Coulomb + van der Waals), atomic charges and atomic densities. The contributions of these features in prediction accuracies are similar as also noted in Figure 3B, suggesting redundancies in features employed. Subsequently, we tested our model’s performance by excluding seemingly redundant features, such as atomic densities and charges from the feature set (Figure 3D). Although minimal, the contribution of each of the structural features holds value in the overall performance. Significantly, solvent accessibility and PSSMs show considerable impact on the model’s performance.

#### 3.2.2 Comparison with state-of-the-art resources

**FIGURE 4:**
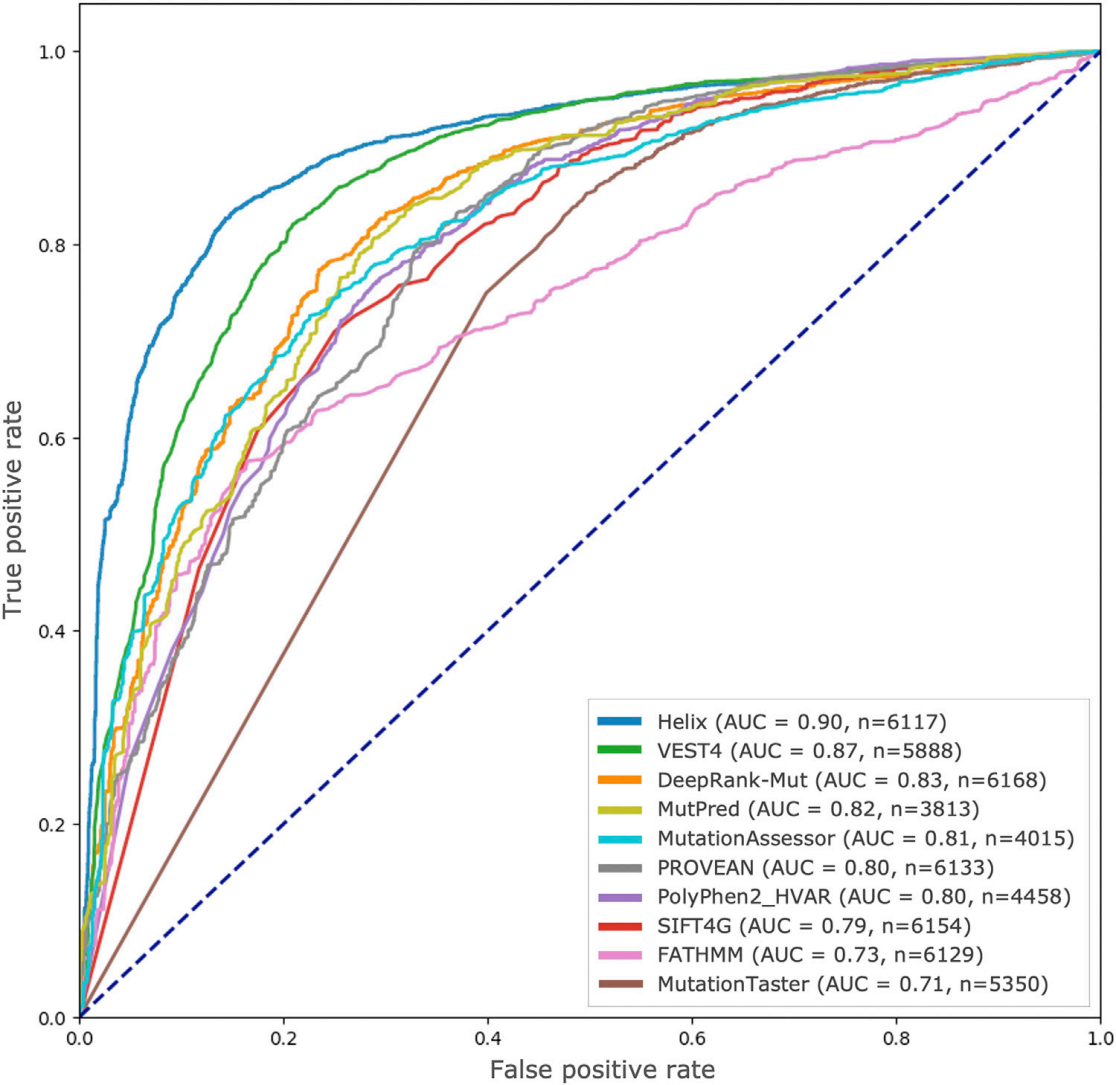
Comparison with other state-of-the-art resources. ROC curves drawn from scores generated by various pathogenicity predictors, including DeepRank-Mut, are shown based on the test variants available for each predictor in dbNSFP.

We used precomputed pathogenicity scores of 8 algorithms from dbNSFP database as well as scores from the Helix for the test dataset used in the study. In the case of PolyPhen2, we used scores from the HumVar-trained models as recommended by the authors for the purpose of distinguishing variants with drastic functional effects from benign ones (Adzhubei et al., 2013). Figure 4 illustrates the ROC curves drawn from these scores along with those from DeepRank-Mut for the variant predictions available for each algorithm. While the performance of our approach is seemingly comparable to other widely-used resources that incorporate sequence conservation and structural features, such as MutPred (Li et al., 2009) and PolyPhen2 (Adzhubei et al., 2010), it must be noted that the available variant predictions for these tools constitute 62% and 72% of the total test set, respectively (*n* in Figure 4, Supplementary Table S1). Both these ML-based tools incorporate several handcrafted features, aside from sequence conservation, including secondary structural assignments, normalized B-factors, and various annotations of functional sites; the only overlapping features with DeepRank-Mut being SASA and sequence conservation. Helix, built on proprietary structure-based sequence alignments (Kuipers et al., 2010; Vroling and Heijl, 2021), and VEST4, a variant prioritization tool that explores enrichment of functional variants across disease exomes (Carter et al., 2013), were notably the top performers.

### 3.3 3D-CNNs appear less powered to identify outcome of solvent-exposed variants

We examined our model’s predictive ability by analyzing missense variants in the test-set that were consistently predicted incorrectly across all 10 folds. We explored the aspects that promoted incorrect classification. A total of 2,883 missense variants were found to be incorrectly classified across the cross-validation experiments, of which more than half (1,732) consisted of misclassified pathogenic variants. We computed relative solvent accessibilities (RSA) for each variant residue, by dividing their absolute solvent accessibilities in Å^2^ by their maximum allowed solvent accessibilities obtained from Rost and Sander (Rost and Sander, 1994). Residues were categorized as solvent-exposed if the RSA values were >20% and buried if below 20%. Using these a substantial proportion of the misclassified pathogenic variants was found to be solvent-exposed (Supplementary Table S2).

**FIGURE 5:**
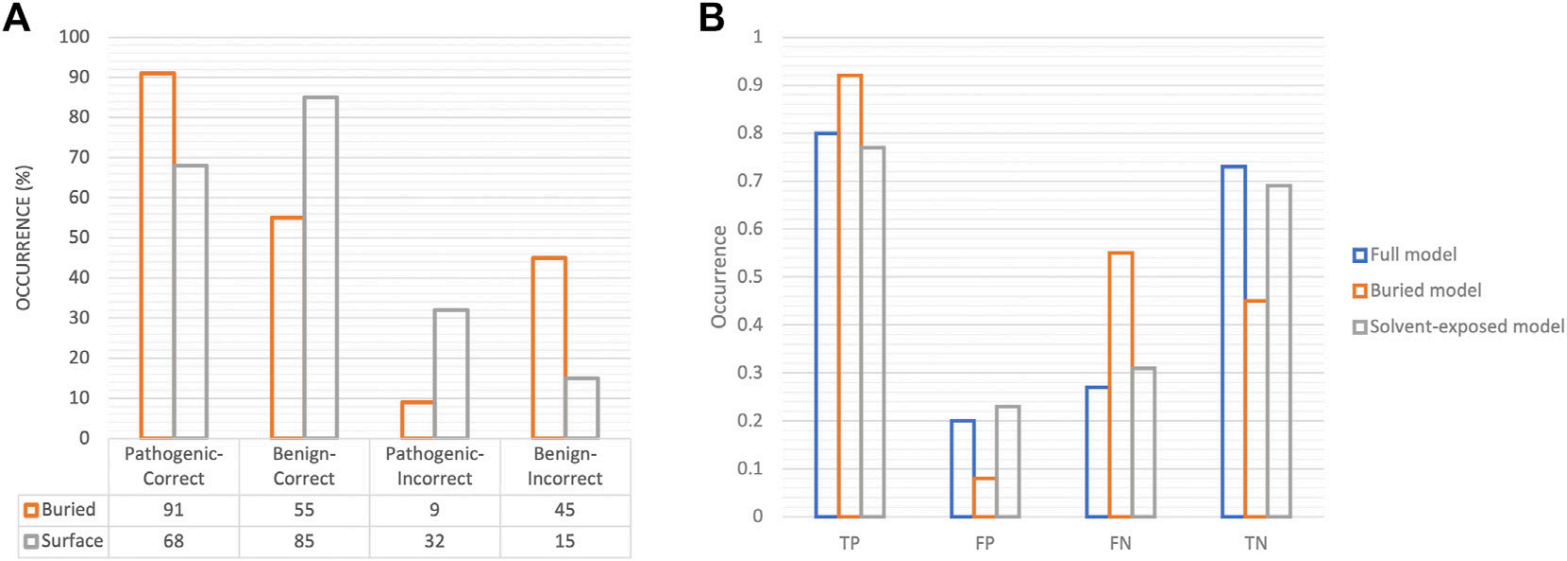
Association of solvent accessibility of variants in prediction outcomes. **(A)** Bar charts for correctly classified and misclassified variants with respect to their solvent accessibility are shown. **(B)** The performance metrics on test data in terms of TP, FP, FN, TN are depicted as bar charts for models trained on all variants (full model), on only buried variants (buried model) and on solvent-exposed variants alone (solvent-exposed model). The proportion of true positives, i.e., pathogenic variants in the model trained on buried variants is notably high.

We constructed 2 × 2 contingency tables based on the correct and incorrect classifications with respect to solvent accessibility of the associated variants. Figure 5 illustrates the role of solvent accessibility in the predicted outcomes. The misclassified variants pertained to solvent-exposed pathogenic variants and buried benign variants (Figure 5A, odds ratio = 0.27). That we are relatively successful in predicting pathogenicity in buried variants is consistent with the notion of buried enrichment of pathogenic variants (Iqbal et al., 2020; Savojardo et al., 2020). The distribution of raw atom-level solvent accessibility values across benign and pathogenic classes calculated in our approach is illustrated in Supplementary Figure S9. Two reasons for the quality of the predictions could be postulated: a) considering the contribution of SASA in the model’s performance, it is likely that the model is unable to generalize on missense variants that fall outside the purview of typical SASA distribution observed in benign and pathogenic variants, or b) the 3D input grids for solvent-exposed missense variants are sparsely populated which leads to a lack of discernible patterns/features for the model to learn from.

We created separate training subsets of buried and solvent-exposed variants to understand 3D-CNN’s generalizability to either subset. We observed that the predictions on pathogenic variants improved with the model trained on buried missense variants alone, however, this model misclassified much of the benign variants, whereas the model trained on solvent-exposed variants alone showed a performance comparable to that of the full model trained on all variants (Figure 5B; Supplementary Figure S5). It is possible that the presence of a large proportion of solvent-exposed variants in our training data may have impacted the performance (Supplementary Figure S9). Furthermore, to assess whether sparsity of 3D grids of solvent-exposed variants affected the model’s performance, we calculated the ratio of solvent (void) voxels to atom-contained (non-void) voxels in the 3D grids in test dataset and compared the distribution of these ratios against the corresponding pathogenic and benign prediction scores. We find no correlation for pathogenic variants (Pearson’s *r* = −0.09), while we find that the presence of void voxels is weakly indicative of correct classifications for benign variants (Pearson’s *r* = −0.24) (Supplementary Figure S10). This overall suggests that grid sparsity has weak effect on the correct classification of benign variants, whereas the incorrect classifications of solvent-exposed pathogenic variants is possibly due to other reasons, such as lack of function-specific features, and/or incomplete knowledge of their interaction partners.

Since data augmentation and feature normalization strategies, typically used to circumvent lack of generalizability and potential biases, are already incorporated in our approach we experimented with inclusion of other structural features: secondary structural content and normalized B-factors. The premise behind use of secondary structural content was based on the report by Abrusán and Marsh (Abrusán and Marsh, 2016), who showed differences in the ability of alpha helices and beta strands to tolerate mutations. Secondary structural assignments for protein structures were obtained from our in-house database (DSSP v.3.1.4) (Kabsch and Sander, 1983), and were stored as one-hot encoded features in 3D grids. B-factors or temperature factors are obtained from X-ray crystallography experiments that indicate atomic flexibility in the protein’s crystalline state, and are known to correlate with flexible regions of the protein. Based on the earlier reports of active/functional sites associated with lower B-factors as compared to non-functional residues (Sun et al., 2019), we used normalized B-factors as a feature to potentially capture such differences. However, the two additional features did not serve as strong determinants of pathogenicity (Supplementary Figure S6). The relatively low quality of predictions for solvent-exposed pathogenic variants and buried benign variants could be due to lack of function-specific features.

### 3.4 Success of pathogenicity prediction depends on underlying disease mechanisms

We further investigated DeepRank-Mut’s generalizability with respect to mutation mechanisms. Most available pathogenicity predictors do not make a distinction between different types of mutation mechanisms such as loss-of-function (LoF) or gain-of-function (GoF), that are often linked to mode of inheritance. LoFs are function-disrupting mutations that usually cause damage to protein structures and are straightforward to comprehend and identify, as they are generally not tolerated at sites of high structural and/or functional importance, and lead to degradation of the protein. In contrast, GoFs exhibit milder effects on protein stability while giving rise to altered protein functions that lead to diseases (Gerasimavicius et al., 2022). In terms of mode of inheritance, autosomal recessive (AR) diseases are predominantly linked to LoFs, while autosomal dominant (AD) diseases manifest through mechanisms such as GoFs, dominant-negative mutations (DN) as well as through LoFs, i.e., haploin sufficiency (Veitia et al., 2018).

**FIGURE 6:**
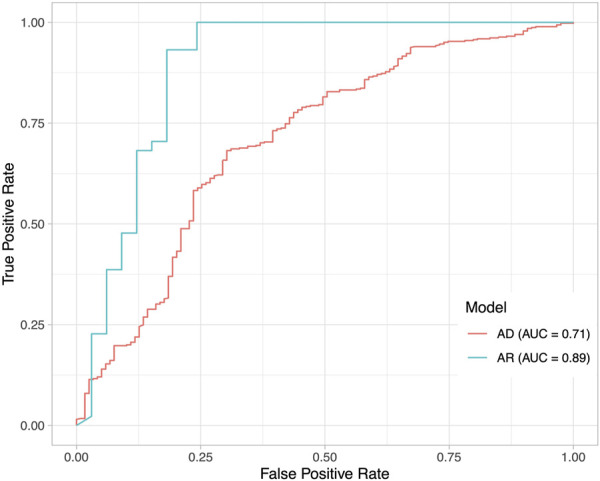
Impact of underlying disease mechanisms on pathogenicity predictions. Performance of DeepRank-Mut on two datasets that are divided based on mode of inheritance. ROC curves are drawn for scores generated from the model tested on variants with AD inheritance (*n* = 585), and from the model tested on variants with AR inheritance (*n* = 77). The AUC values are markedly different between the two datasets as depicted. It must be noted that the predictions are made for those variants that could be mapped to protein crystal structures.

**FIGURE 7:**
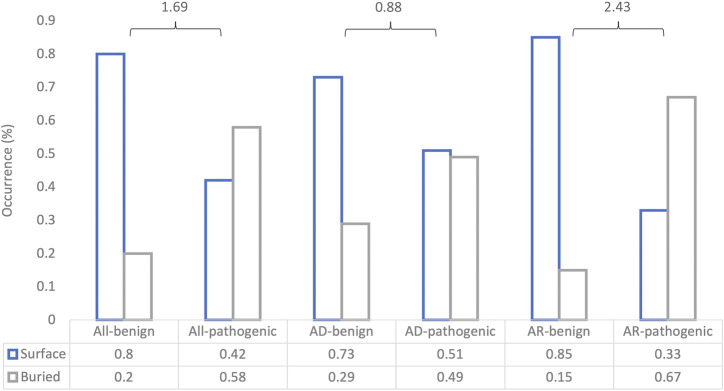
Association of missense variants across predictions on different test datasets with solvent accessibility. The bar plot shows the proportion of surface-exposed and buried missense variants in each of the binary outcomes for each of the datasets. “All” denotes all input variants, AD denotes mutations with autosomal dominant inheritance, and AR denotes mutations with AR inheritance. The log-odds ratio is calculated for each case to determine the strength of association between the binary feature (buried or surface-exposed) and the binary outcome (benign or pathogenic).

To understand how DeepRank-Mut generalizes on distinct modes of inheritance of pathogenic variants, we split our test datasets into variants with AD inheritance (*n* = 1,363; 550 benign, 813 pathogenic) and variants with AR inheritance (*n* = 563; 244 benign, 319 pathogenic), based on information obtained from ClinVar (Landrum et al., 2018). Only a smaller subset could be mapped to crystal structures: 585 structures mapped to 515 AD variants, and 77 structures mapped to 132 variants. We did not filter the AD dataset further to segregate mutations into haploinsufficient genes (LoFs) and non-LoFs (GoFs, DNs), due to lack of detailed annotations of non-LoFs in ClinVar. However, it is worth noting that mutations in the AD dataset could consist of higher proportion of LoFs than non-LoFs due to smaller mutational target for non-LoFs, i.e., fewer mutations alter protein function than disrupt it. Figure 6 illustrates a marked difference in the model’s performance between the two datasets, suggesting dependence on underlying effects of the variant on the protein. It is apparent from the figure that our model is able to generalize AR mutations (LoFs) better than AD mutations (LoFs and non-LoFs). Details on the pathogenicity predictions obtained for AD and AR datasets, are provided in Supplementary Table S3. To further examine our relative success in correctly classifying buried pathogenic variants and AR variants we analyzed the distribution of solvent accessibility in the AD and AR datasets. Interestingly, the typical distribution of solvent-exposed benign variants and buried pathogenic variants was found to be more pronounced in AR datasets than in the AD datasets (Figure 7), explaining the relative success of our model in distinguishing LoF mutations from benign (Supplementary Figure S7, MCC = 0.67).

## 4 Discussion

Numerous efforts in the last decade have aided in the general understanding of effects of disease-causing mutations on the biophysical characteristics of proteins-including protein stability, dynamics, and protein-protein interactions (Kucukkal et al., 2015; Iqbal et al., 2020). It has been observed that pathogenic mutations are often associated with changes in local hydrogen-bonding network, electrostatic interactions, and overall side-chain geometry (Kucukkal et al., 2015). Although this knowledge has helped in the advancement of variant effect predictors that integrate various information on top of sequence-based features, the accurate prediction of a functional outcome of a missense variant is often fraught with challenges that we partly bring forth in this study.

We describe DeepRank-Mut, a structure-guided approach that leverages properties in the local variant neighborhood and uses 3D-CNNs to draw relationships between the spatially proximal features to distinguish pathogenic missense variants from benign. Our approach is robust to rotational variations, as we account for different orientations of a given variant environment through data augmentation steps. We did not experiment with larger augmentations due to large computational costs incurred. The performance of DeepRank-Mut was found to be comparable with other widely used predictors, such as PolyPhen2 which employs classical ML algorithm and relies on handcrafted features. Our investigations into the generalizability of our model revealed aspects that could be of interest to those who adopt deep learning techniques in structure-based variant effect predictions.

We find that the evolutionary information (PSSM profile) of the variant neighborhood captures patterns in the 3D structural context of variant sites better than the individual structural properties themselves. In contrast, inclusion of variant site-specific conservation scores alone, devoid of the 3D context, render the 3D-CNN model myopic thereby affecting the overall predictive ability. This finding is of considerable significance as it shows that the model potentially draws context dependence in terms of evolutionarily coupled residues. Pairs of residues under structural and functional constraints can exhibit strong inter-residue correlations, and thus coevolve (de Juan et al., 2013). Such a property has been shown to be useful in capturing effects of genetic variations (Hopf et al., 2017). Without explicitly modeling such inter-residue correlations, the performance of our model trained only on the PSSM profile of the neighborhood illustrates the utility of 3D-CNNs in capturing complex relationships between residues. This is further strengthened by the leave-one-feature-out analysis, where exclusion of seemingly redundant features from the model affected its performance.

Solvent accessible surface area was identified as the second most important feature that contributed to the predictor accuracies. Considering earlier reports on the enrichment of solvent-exposed missense variants in populations and enrichment of pathogenic variants in the hydrophobic core of proteins (Iqbal et al., 2020; Savojardo et al., 2020), we sought to explore their distribution in missense variants which were consistently misclassified across our datasets. We note that a significant proportion of misclassified pathogenic variants were found to be solvent-exposed, which raises the question whether our model loses generalizability while prioritizing buried pathogenic variants. Our experiments with models separately trained on buried and solvent-exposed missense variants yielded interesting results. The buried model could correctly identify pathogenic variants, even those that are solvent-exposed, while misclassifying a significant proportion of benign variants. The solvent-exposed model, on the other hand, showed similar performance in comparison to the original full model trained on all variants. These findings necessitate incorporating function-specific features or use of other suitable representations of protein structures, such as graphs, to adequately capture the underlying differences within pathogenic missense variants. Achieving high classification scores on solvent-exposed variants do pose a challenge, yet may be overcome with the following strategies: a) ensemble learning, combining multiple models trained on different feature sets related to solvent-exposed variants, such as ligand binding sites or phosphorylation sites; b) active learning, iteratively selecting the most informative solvent-exposed variants for labeling and training the model; or c) self-supervised learning, training the model to predict masked residues. Moreover, it is also possible that the solvent-exposed pathogenic variant site is a part of a larger assembly or participates in protein-protein interactions, an aspect not considered in this study. Use of full protein complex structures for pathogenic variants, wherever applicable, or features that indicate their role in function could help improve classifications (Gerasimavicius et al., 2022). Overall, we find that the two main features: evolutionary information of residues in the variant neighborhood and solvent accessibilities sufficiently capture most of the important traits around variant sites.

Consideration of disease mechanisms appears to be crucial in the quality of pathogenicity predictions, as exemplified in our study. Our approach could generalize on mutations linked to AR inheritance better than the mutations linked to AD inheritance, corroborating results from an earlier study by Gerasimavicius et al. (2022). This finding is primarily due to the underlying mechanisms of mutations where protein destabilizing LoFs, often associated with AR diseases, are more straightforward to identify than non-LoFs which tend to have milder impacts on protein stability. Moreover, distribution of solvent accessibility of variants was suggestive of notable differences in the proportion of buried and solvent-exposed pathogenic variants, across the datasets. The overall performance of AR datasets over AD dataset is potentially due to two plausible reasons: a) feature representations are sufficiently able to distinguish LoFs from benign, and not non-LoFs from benign and b) limited amount of data on variants with non-LoF mechanisms. Both these postulates hold true considering the damaging effects on protein structure caused by LoFs that are relatively straightforward to discern (Gerasimavicius et al., 2022), and considering the total size of missense variants with non-LoF mechanisms (GoF and DN) mapped onto protein structures (*n* = 972), which is insufficient for training using deep neural networks. Since we did not segregate the AD dataset further into non-LoFs (GoFs, DNs) and LoFs, i.e., mutations in haploinsufficient genes, it is not apparent how the PSSM profile of residues in a variant environment and their solvent accessibility impact the predictions made. Nevertheless, our analysis underscores the necessity of incorporating features related to non-LoFs in improving pathogenicity predictions. This can be achieved through scrutiny and inclusion of gene-level and protein-level features specific to each of the mutation mechanisms in question, as documented by Sevim Bayrak et al. (2021). In addition, proteins in both AD and AR datasets reportedly show significant differences in functional class prevalence (Gerasimavicius et al., 2022), necessitating function-specific analysis to delineate characteristics of the disease mechanisms of mutations (Iqbal et al., 2020).

Our current method does not include explicit modeling of mutations into the protein structure, nor inclusion of protein dynamics, an inherent property linked to protein function. Indeed, inclusion of such details can aid in the recognition of the extent of mutation-induced changes in intra-protein structural contacts, as well as changes in thermodynamic stability (Rodrigues et al., 2018). In combination with other relevant features, these may provide considerable insights into understanding different effects across different mutation types, even with limited protein structural data. While we acknowledge the limitations of training our model on static protein microenvironments, we understand that more features may not necessarily imply better performance with neural networks. With suitable representations of protein structures (graphs) and information on protein dynamics it is important to address fundamental problems, such as predicting functional sites (Chiang et al., 2022) or predicting structurally important sites to further our understanding of model-driven approaches. This can help gauge utility of protein dynamics-informed or physics-informed graph representations in predicting variant pathogenicity.

To summarize, we have described a structure-guided approach to predict functional outcomes of missense variants using 3D-CNNs. We analyze and demonstrate the contribution of different features on the predictive ability of the neural network. Of particular note is the influence of evolutionary information of the variant neighborhood and their solvent accessibilities in determining variant pathogenicity. We further provide detailed assessment of our model’s generalizability on distinct mechanisms of mutations, which presents a complex but critical challenge in improving pathogenicity predictions. Our analysis presents lessons to consider when using model-driven approaches to address questions in structure-guided predictions of variant pathogenicity.

## Data Availability

The original contributions presented in the study are included in the article/Supplementary Material, further inquiries can be dirrected to the corresponding author. The source code and documentation of DeepRank-Mut are available at https://github.com/DeepRank/DeepRank-Mut/.
