# Supplementary material for: Understanding structure-guided variant effect predictions using 3D convolutional neural networks
Source: Front Mol Biosci. 2023 Jul 5;10:1204157. doi: 10.3389/fmolb.2023.1204157 (PMC10354367; doi:10.3389/fmolb.2023.1204157)
Supplement: Supplementary file 4 [file DataSheet1.docx]

# Supplementary Methods

## Details on feature calculations

The feature calculations are performed similar to our parent framework, DeepRank (Renaud et al., 2021). This section details calculations for pairwise Coulomb and van der Waals potentials between atoms of the residue at variant position, and the atoms of residues around it.

Pairwise Coulomb potentials are calculated between the residue at variant site with *I* set of atoms, and *J* set of atoms in residues within the variant environment (10Å radius). For a given atom *i* in *I*, the total electrostatic energy *E_c,i_* is given by the sum of all pairwise interactions with atoms in *J*:

$$E_{c,i}=\sum_{j\in J} E_{c,ij}$$

Similarly, the total electrostatic energy on atom *j* is given by:

$$E_{c,j}=\sum_{i\in I} E_{c,ij}$$

Electrostatic energy *E_c,ij_* between non-bonded atoms *i* and *j* is given by:

(1)

$$E_{c,ij}= \frac{q_{i}q_{j}\frac{C}{\varepsilon_{0}}}{r_{ij}} f_{e}$$

where:

$$f_{e}=1-\frac{r_{ij}}{r_{0}}$$

*r_0_* = 8.5Å

*C* = 332.0636

𝜀_0_ = 1.0

The electrostatic energy *E_c,ij_* for bonded atoms (< 2.2Å) is set to 0. The atomic charges *q_i_* and *q_j_* are based on the OPLS forcefield similar to the parent framework.

Pairwise van der Waals potentials are calculated between the residue at variant site with *I* set of atoms, and *J* set of atoms in residues within the variant environment. Similar to the electrostatic energy calculations, for a given atom *i* in *I*, the total van der Waals energy *E_vdw,I_* is given by the sum of all pairwise interactions with atoms in *J,* while *E_vdw,j_* represents total van der Waals energy on atom *j:*

$$E_{vdw,i}=\sum_{j\in J} E_{vdw,ij}$$

$$E_{vdw,j}=\sum_{i\in I} E_{vdw,ij}$$

The van der Waals potential is approximated by Lennard-Jones potential (Jones and Chapman, 1997; Lennard-Jones, 1931) between non-bonded atoms *i* and *j.* This interaction energy separated by three inter-atomic bonds, is given by:

$$E_{vdw,ij}=4\varepsilon_{ij,intra}\left( \left( \frac{\sigma_{ij,intra}}{r_{ij}} \right)^{12}- \left( \frac{\sigma_{ij,intra}}{r_{ij}} \right)^{6} \right) f_{vdw,ij}$$

while the interaction energy separated by four or more inter-atomic bonds is given by:

(3)

(2)

$$E_{vdw,ij}=4\varepsilon_{ij,inter}\left( \left( \frac{\sigma_{ij,inter}}{r_{ij}} \right)^{12}- \left( \frac{\sigma_{ij,inter}}{r_{ij}} \right)^{6} \right) f_{vdw,ij}$$

where:

$$\varepsilon_{ij, intra}= \sqrt{\varepsilon_{i,intra}\varepsilon_{j,intra}}$$

$$\varepsilon_{ij, inter}= \sqrt{\varepsilon_{i,inter}\varepsilon_{j,inter}}$$

$$\sigma_{ij,intra}=0.5\left( \sigma_{i, intra}+ \sigma_{j,intra} \right)$$

$$\sigma_{ij,inter}=0.5\left( \sigma_{i, inter}+ \sigma_{j,inter} \right)$$

Also, if *r_ij_* < *r_on_* :

$$f_{vdw,ij}=1.0$$

else if *r_ij_* > *r_off_* :

$$f_{vdw,ij}=0.0$$

else:

$$f_{vdw,ij}= \frac{\left( r_{off}^{2}-r_{ij}^{2} \right)^{2} \left( r_{off}^{2}- r_{ij}^{2}-3\left( r_{on}^{2}- r_{ij}^{2} \right) \right)}{\left( r_{off}^{2}- r_{on}^{2} \right)^{3}}$$

where *r_on_* = 6.5Å and *r_off_* = 10.0Å

The parameters 𝜀 and 𝜎 are specific to atom types and is retained from the parent DeepRank framework.

## Distributed Data Processing (DDP)

Due to large volumes of data for preprocessing tasks it was essential to adapt DeepRank-Mut’s algorithm to optimize for GPU usage, which was achieved in the feature mapping steps. This optimization allowed for testing broader range of variants that was previously difficult due to computational constraints. The optimized framework we developed was used for this study alone, it is not available in the program’s github repository.

Centralized computing systems are not always suitable for handling large volumes of data due to their limited capacity. As the size of data increases, the processing time also increases, leading to performance degradation and potential system crashes. To overcome the limitations, we employed distributed data processing, which divided the data processing tasks into smaller parts to be handled by multiple nodes in parallel. This approach enabled faster processing times, greater scalability and fault tolerance.

The framework utilized three servers, with one acting as the main node (Supplementary Figure S1). The main node housed a DDP-Delegator container, which was responsible for communicating with the DDP-Worker containers. The DDP-Delegator container was designed to distribute input data to all DDP-Workers and to assign new jobs to available workers once they had a task is complete. The DDP-Worker containers ran an API that received jobs from the DDP-Delegator. Once a job was received, the worker container would initiate preprocessing of the data using the existing DeepRank-Mut preprocessing functionalities. After completion of the task, the preprocessed data was sent to the DDP-Collector container, which compiled the job and stored the preprocessed data in a HDF5-file for further use in DeepRank-Mut’s training module.

## Hyperparameter tuning

We employed several iterations of experiments to identify the optimal settings for our 3D-CNN model. We started from the default parameters used in the parent DeepRank and tested for number of augmentations (Supplementary Figure S3), optimal grid sizes (Supplementary Figure S4), and optimal number of convolutional layers and number of max pooling layers (Supplementary Figure S8),

# Supplementary Figures and Tables

## Supplementary Figures


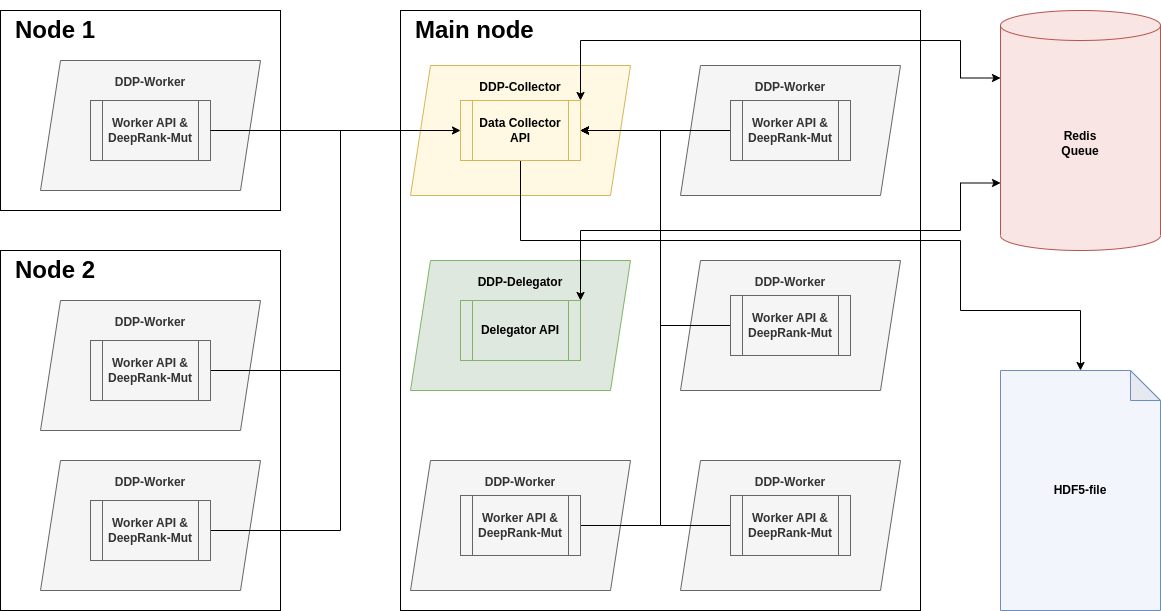


**Supplementary Figure S1.** Architecture of DDP. The diagram illustrates a Distributed Data Processing system which consists of multiple nodes that act as servers, each running specialized DDP- Delegator, DDP-Worker, or DDP- Collector containers. To facilitate communication between the nodes, a Redis Queue is utilized, enabling the DDP-Delegator and DDP-Collector to exchange information on the status of jobs. As a DDP-Worker completes a task, it sends the results to the DDP-Collector, for compilation and storage as a HDF5-file.


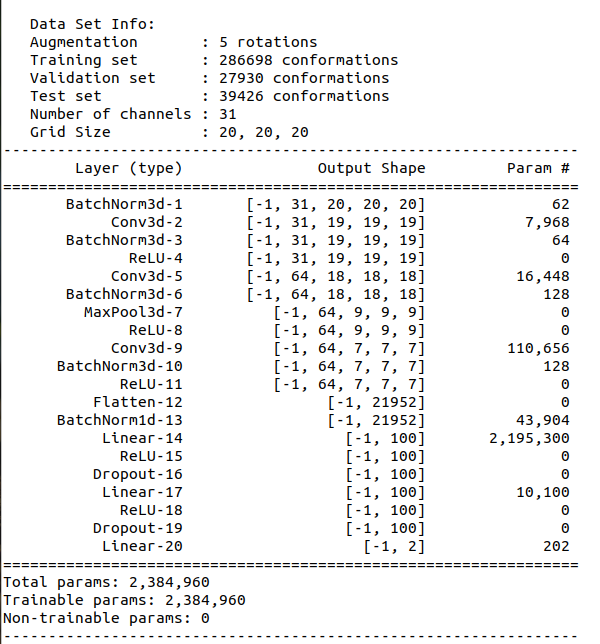


**Supplementary Figure S2.** Schema of the neural network architecture and sizes of the datasets used are shown. The data pertains to training, validation and test sets from a single fold. Due to the data augmentation step, the total number of variant instances increase by a factor 6 (original grid + 5 rotations).


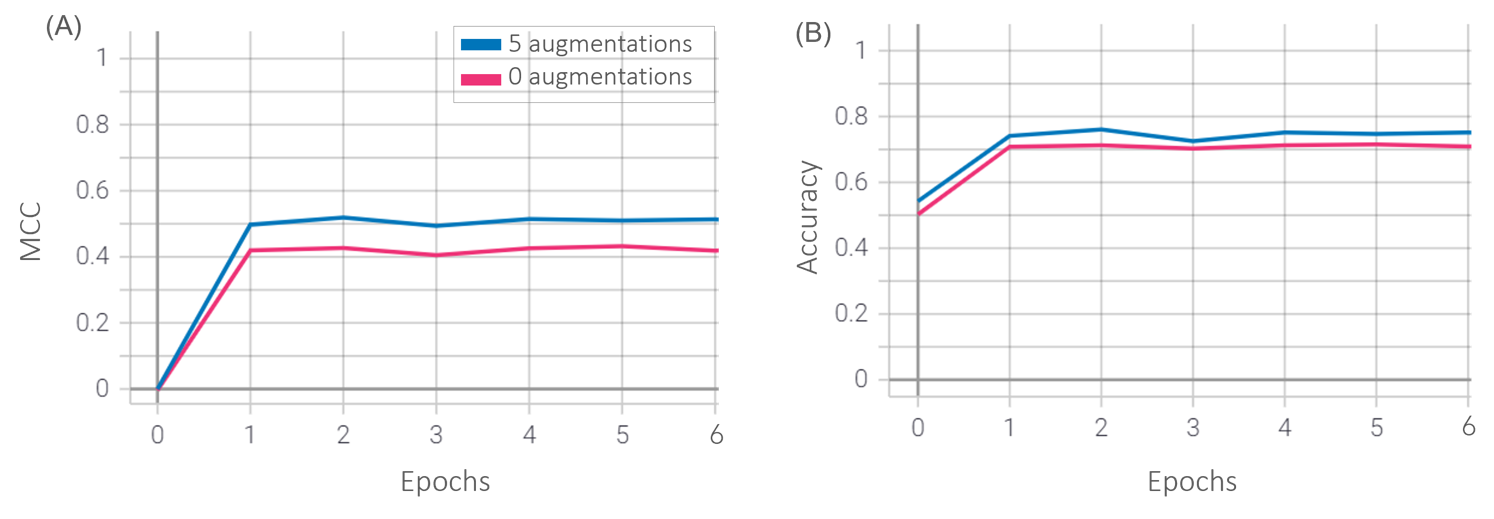


**Supplementary Figure S3.** Data augmentation strategy helps improve performance of the classifier. Models trained with augmentations (blue) and without (pink) augmentations exhibit differences in MCC scores A) and in the accuracies B). For the dataset without augmentation, the average MCC obtained was 0.41 with a mean accuracy of 0.70. Results are shown for the best-performing fold.


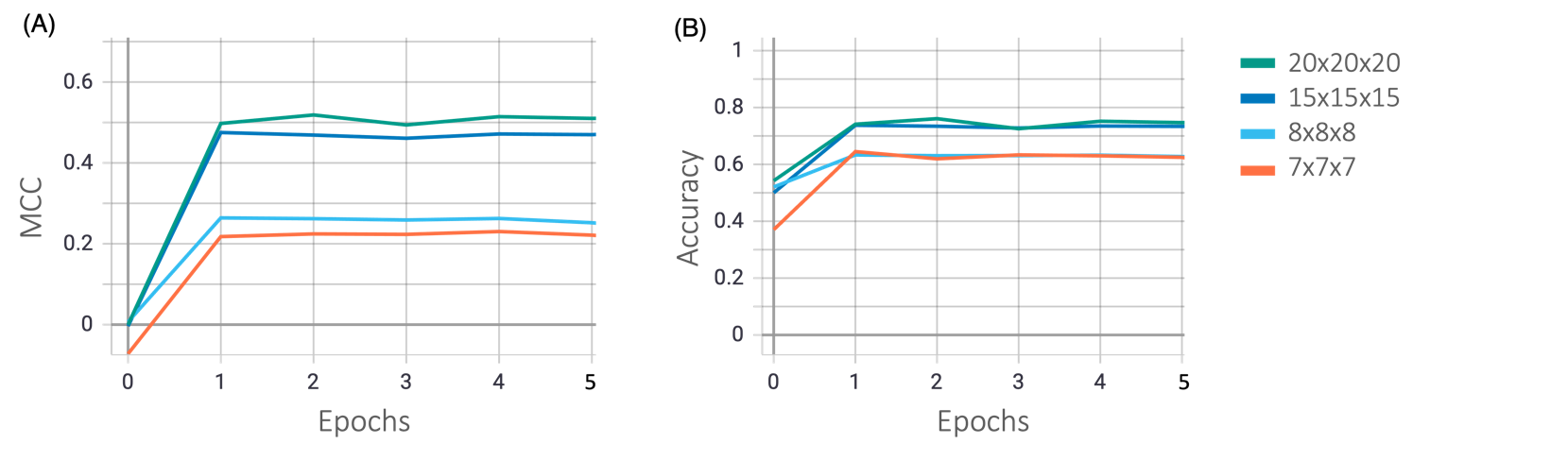


**Supplementary Figure S4.** Effect of grid size on model’s performance. Features extracted using smaller grid sizes 7Å (orange), 8Å (light blue) and 15Å (dark blue) affected the performance of our model, in comparisonwith model built using grid box of size 20Å (green) centered at the variant. Results are shown for the best-performing fold alone.


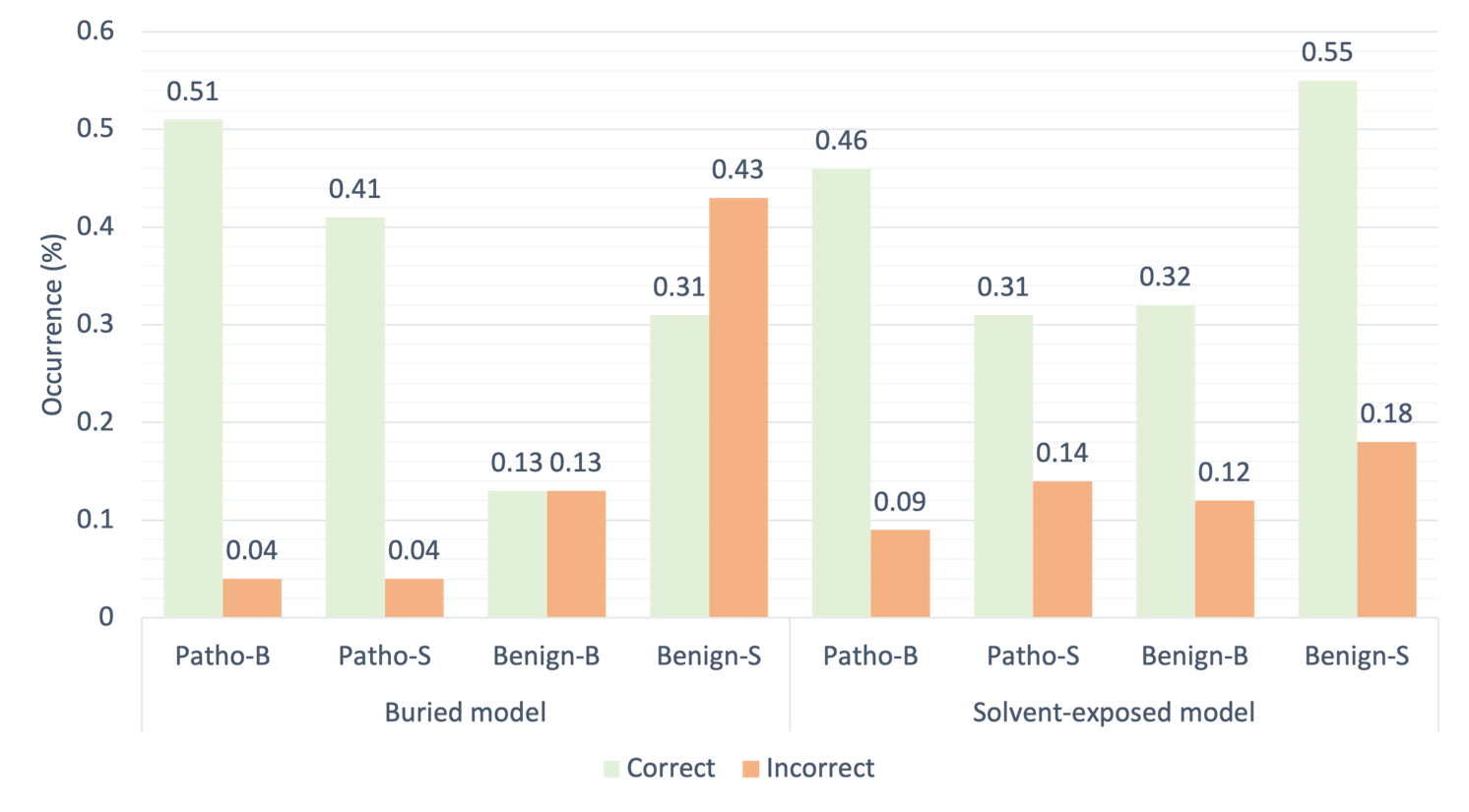


**Supplementary Figure S5.** Impact of solvent accessibilities of variants on model’s generalizability. Distribution of solvent accessibilities of variants between correctly classified and misclassified variants across the two experimental models are shown. The terminologies in x-axis are as follows: Patho-B, pathogenic buried variants; Patho-S, pathogenic solvent-exposed variants; Benign-B, benign buried variants; and Benign-S, benign solvent-exposed variants.


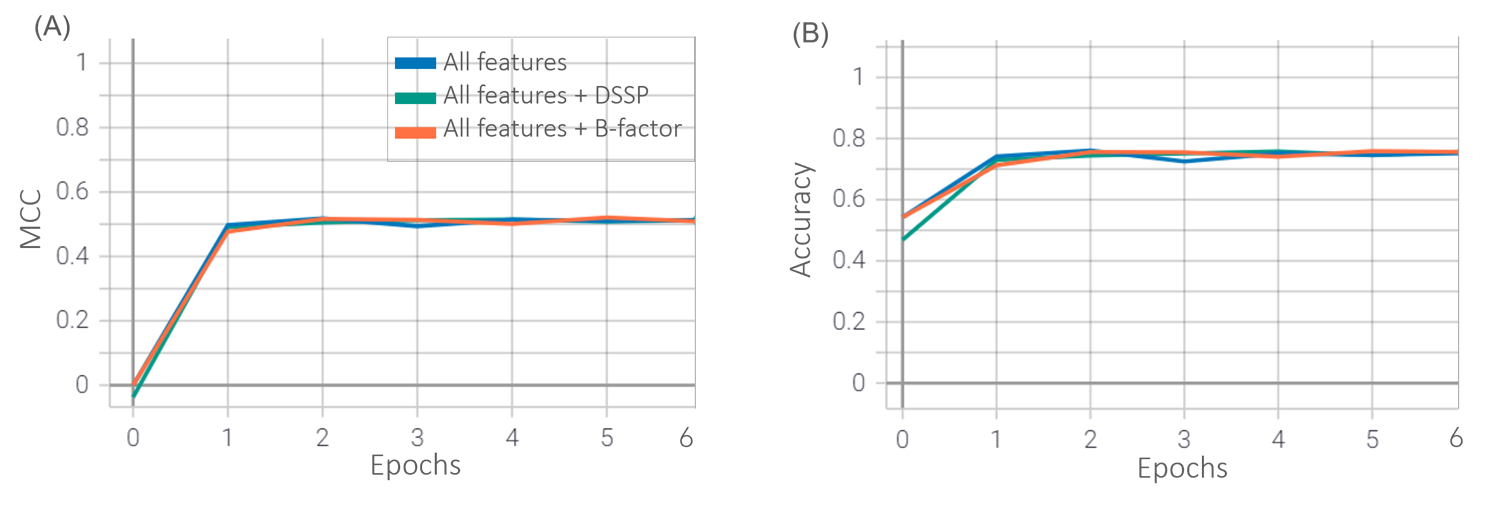


**Supplementary Figure S6.** Experiments with two additional structural features. Models trained on all 31 features (blue), 31 features + secondary structural content (green), and 31 features + normalized B-factors (orange) showed negligible difference in performance on test data. MCC A) and accuracies B) for the three models during training are shown over 6 epochs for the best-performing fold.


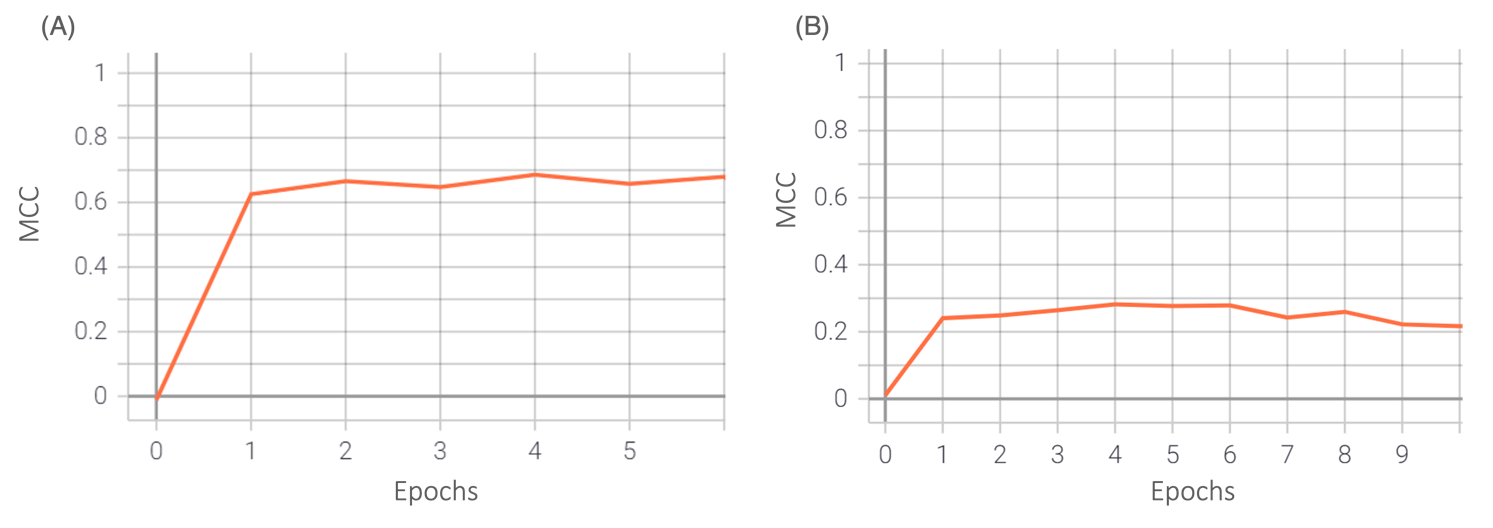


**Supplementary Figure S7.** Differences in performance of DeepRank-Mut tested on A) mutations inherited in an autosomal recessive manner (AR) (average MCC= 0.67 over 6 epochs), and B) mutations inherited in an autosomal dominant manner (AD) (average MCC= 0.28 over 10 epochs).


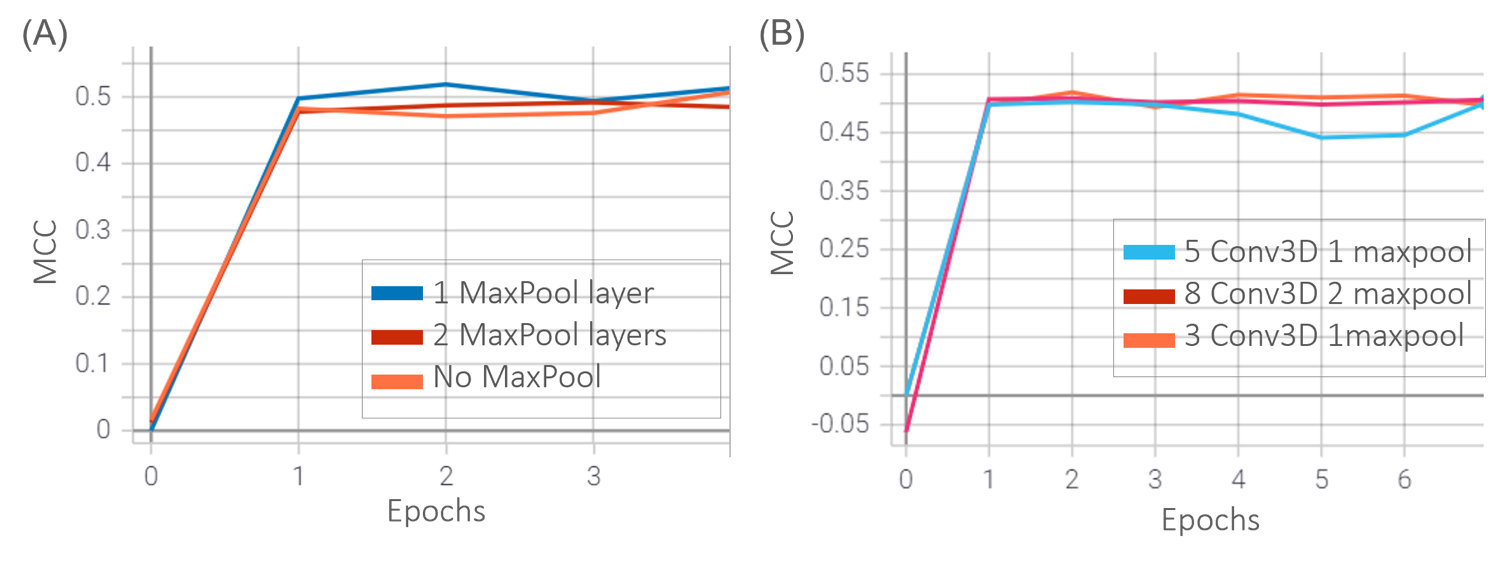


**Supplementary Figure S8.** Tuning for optimal number of layers in DeepRank-Mut’s architecture. A) Performance of models built with 3 convolutional layers and single maxpool layer (dark blue), 3 convolutional layers and 2 maxpools (red), and 3 convolutional layers without maxpool (orange) are depicted. B) Performance of models built with 3 convolutional layers (orange), 5 convolutions (light blue) and 8 convolutional layers with 2 maxpools (red) are shown. From these experiments, we decided on deploying 3 convolutions and 1 maxpool layer in our final network.

**
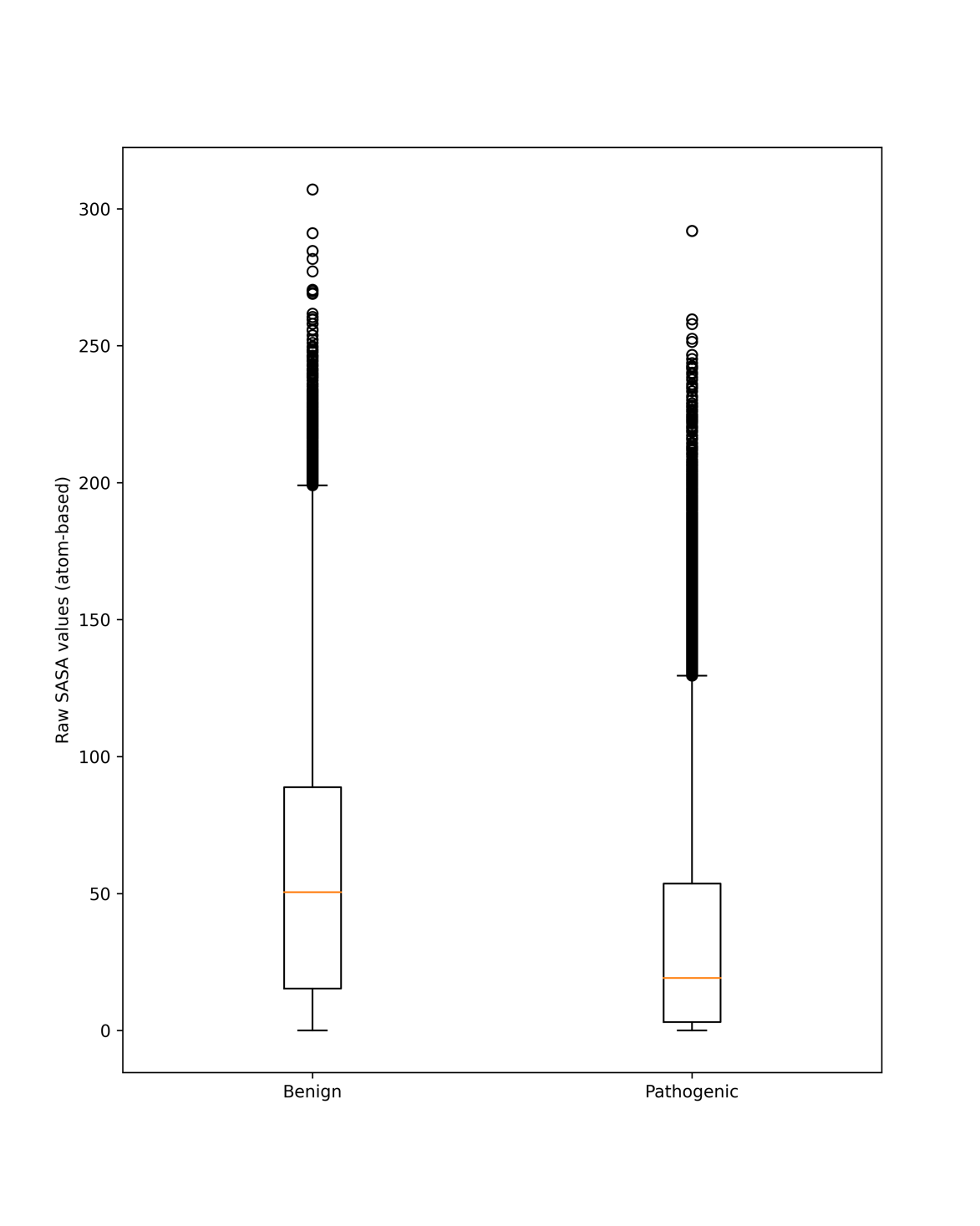
**

**Supplementary Figure S9.** Boxplots showing raw atom-level solvent accessibility scores, obtained from FreeSASA, across pathogenic and benign variants in the training dataset. Values in y-axis are in Å^2^.


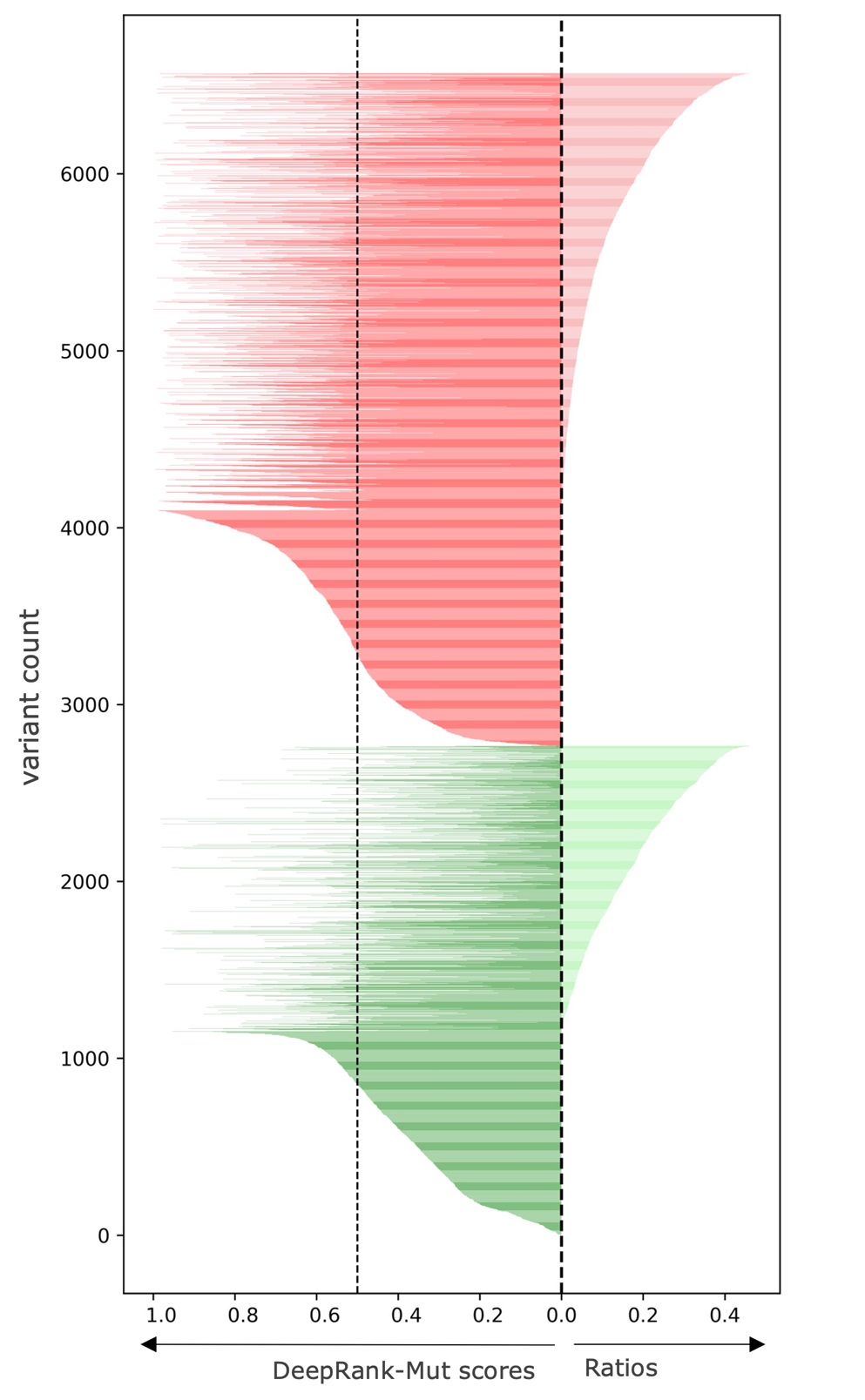


**Supplementary Figure S10.** Bidirectional bar chart showing distribution of ratios of void over non-void voxels (right) in 3D grids in the test dataset (*n*= 6571) and their associated predicted scores from DeepRank-Mut (left). The colors denote true labels for pathogenic (red) and benign (green) variants. Since each 3D grid comprises of 31 feature channels, we obtain 31 ratios per grid; we take the median value for the plot. The vertical line at *x*= 0.5 (left) denotes the cutoff value used in DeepRank-Mut scores; 0 is benign and 1 is pathogenic.

## Supplementary Tables

**Supplementary Table S1:** Details of prediction scores on test set from various pathogenicity predictors.

**Supplementary Table S2:** Details of variants consistently misclassified across 10 folds.

**Supplementary Table S3:** Details on the pathogenicity predictions for AR and AD test sets using DeepRank-Mut.

# References

Jones, J.E., Chapman, S., 1997. On the determination of molecular fields.—I. From the variation of the viscosity of a gas with temperature. Proceedings of the Royal Society of London. Series A, Containing Papers of a Mathematical and Physical Character 106, 441–462. https://doi.org/10.1098/rspa.1924.0081

Lennard-Jones, J.E., 1931. Cohesion. Proc. Phys. Soc. 43, 461. https://doi.org/10.1088/0959-5309/43/5/301

Renaud, N., Geng, C., Georgievska, S., Ambrosetti, F., Ridder, L., Marzella, D.F., Réau, M.F., Bonvin, A.M.J.J., Xue, L.C., 2021. DeepRank: a deep learning framework for data mining 3D protein-protein interfaces. Nat Commun 12, 7068. https://doi.org/10.1038/s41467-021-27396-0
